# Crosstalk Between Autophagy and Ferroptosis and Its Putative Role in Ischemic Stroke

**DOI:** 10.3389/fncel.2020.577403

**Published:** 2020-10-02

**Authors:** Jie Liu, Zhen-Ni Guo, Xiu-Li Yan, Shuo Huang, Jia-Xin Ren, Yun Luo, Yi Yang

**Affiliations:** ^1^Department of Neurology, Stroke Center & Clinical Trial and Research Center for Stroke, The First Hospital of Jilin University, Changchun, China; ^2^China National Comprehensive Stroke Center, Changchun, China; ^3^Jilin Provincial Key Laboratory of Cerebrovascular Disease, Changchun, China

**Keywords:** autophagy, ferroptosis, cell death, ischemic stroke, iron overload, lipid peroxidation, reactive oxygen species

## Abstract

Autophagy is a conserved process to maintains homeostasis via the degradation of toxic cell contents, which can either promote cell survival or accelerate cellular demise. Ferroptosis is a recently discovered iron-dependent cell death pathway associated with the accumulation of lethal reactive lipid species. In the past few years, an increasing number of studies have suggested the crosstalk between autophagy and ferroptosis. Ischemic stroke is a complex brain disease regulated by several cell death pathways, including autophagy and ferroptosis. However, the potential links between autophagy and ferroptosis in ischemic stroke have not yet been explored. In this review, we briefly overview the mechanisms of ferroptosis and autophagy, as well as their possible connections in ischemic stroke. The elucidation of crosstalk between different cell death pathways may provide insight into new future ischemic stroke therapies.

## Introduction

Stroke is one of the major causes of death and disability worldwide (Haley et al., 2019), and includes two main subtypes: ischemic stroke and hemorrhagic stroke. Ischemic stroke results from a lack of blood supply to the brain, and accounts for approximately 85% of all cases of stroke. Nowadays, several cell death pathways have been identified to be involved in ischemic stroke pathophysiology, including apoptosis, necrosis, and autophagy. Among these, necrosis has been generally regarded as a passive and uncontrolled form of cell death, while more recently, certain types of regulated necrosis have also been found, such as necroptosis, pyroptosis, ferroptosis, parthanatos, and CypD-mediated necrosis. Interventions targeting these specific types of regulatory necrosis have provided new ideas for the treatment of ischemic stroke (Lu et al., 2020).

Autophagy is an evolutionarily conserved process to degrade toxic proteins, damaged organelles, and invading pathogens via the lysosomal pathway. At the molecular level, autophagy is mainly executed by multiple autophagy-related genes (Atg), and is also regulated by a complex signaling network. Autophagy plays an important role in maintaining cellular homeostasis, regulating organism growth, and modulating the development of diseases (Doria et al., 2013). In ischemic stroke, it can either postpone or accelerate cell death, depending on the degree of activation.

Ferroptosis is iron-dependent regulated necrosis associated with excess reactive lipid species, due to accumulated lipid peroxidation. Recently, studies found that ferroptosis also plays an important role in the development of ischemic stroke through influencing iron metabolism or lipid peroxidation, whereas inhibiting ferroptosis successfully reverses ischemic damages (Tuo et al., 2017; Alim et al., 2019; Guan et al., 2019). Furthermore, ferroptosis is significantly distinct from other forms of cell death, including autophagy, in terms of cell morphological characteristics, biological features and so on (Dixon et al., 2012; Klionsky et al., 2016; Xie et al., 2016; Zille et al., 2017). Conventionally, autophagy-dependent cell death is characterized by the formation of autophagosomes, which fuse with lysosomes to form autolysosomes. Differently, ferroptosis is featured by an intact cell membrane and normal nucleus, but shrinking mitochondria with increased membrane densities, reduced even disappeared mitochondria crista and ruptured outer membrane. For detailed information regarding the difference between autophagy and ferroptosis (see Table 1).

**TABLE 1:** Characteristics of autophagy and ferroptosis.

	Autophagy	Ferroptosis
Morphological characteristics	Formation of autophagosomes, a double membrane vesicle containing multiple cytoplasmic contents. The formed autophagosomes fuse with lysosomes to form autolysosomes	Atrophy of mitochondria with increased membrane densities, reduced even disappeared mitochondria crista and ruptured outer membrane, normal nucleus
Development steps	Iron overload, GSH depletion and Gpx4 inactivation, lipid peroxidation, and impaired system Xc-	Initiation and nucleation of autophagosomes, maturation of autophagosomes and fusion of autophagosomes with the lysosome and degradation
Key regulators	Atgs, mTOR, AMPK, BECN1, PI3K, p62, p53, ULK1, TFEB	Positive regulator: SAT1, GLS2, p53, ACSL4, TfR1 Negative regulator: GSH, NRF2, HSPA5, NCOA4, HSPB1, Gpx4, SLC7A11
Common inducers and inhibitors	Inducer: Rapamycin, Brefeldin A, Tunicamycin, starvation media Inhibitor: 3-MA, Bafilomycin A1	Inducer: Erastin, RSL3, Sorafenib, SAS, FIN56, FAC Inhibitor: DFO, Fer-1, Vitamin E, Liproxststatin-1, 2,2-BP, DFA, ciclopirox olamine

**Atgs, autophagy-related genes; AMPK, Adenosine 5’-monophosphate (AMP)-activated protein kinase; BECN1, beclin 1; PI3K, Phosphatidylinositol 3-kinase; TFEB, Transcription factor EB; 3-MA, 3-Methyladenine; GLS2, Glutaminase 2; ACSL4, Acyl-CoA synthetase long chain family member 4; TfR1, Transferrin receptor 1; GSH, glutathione; NRF2, nuclear factor E2 related factor 2; HSPA5, Heat shock 70kDa protein 5; NCOA4, Nuclear receptor coactivator 4; HSPB1, heat shock protein family B (small) member 1; Gpx4, glutathione peroxidase 4; SLC7A11, solute carrier family 7 member 11; RSL3, Ras-selective lethal small molecule 3; SAS, sulfasalazine; FAC, ferric ammonium citrate; DFO, deferoxamine; Fer-1, Ferrostatin-1.**

While autophagy and ferroptosis are mechanistically and morphologically distinct cell death pathways, an increasing number of studies have recently reported significant crosstalk between them (Kang and Tang, 2017; Zhou et al., 2019; Liu et al., 2020). This identification not only favors a deeper understanding of cell death, but also provides new ideas for the regulation of disease and development of therapeutic strategies. In this review, we briefly introduce the mechanisms of autophagy and ferroptosis, as well as pathways that mediate their interactions. On this basis, we further discuss their possible interrelationships in ischemic stroke.

## Autophagy and Ischemic Stroke

### Mechanisms of Autophagy

Autophagy can be divided into three general subtypes: macroautophagy, microautophagy, and chaperone-mediated autophagy (Klionsky et al., 2016). Macroautophagy is a continuous and dynamic process initiated by the formation of autophagosomes, which are double membrane vesicles that contain multiple cytoplasmic components, including damaged organelles and dysfunctional proteins. The formed autophagosomes then fuse with lysosomes to form autolysosomes, and induce the degradation and recycling of cellular components. This process allows cells to maintain homeostasis under stressful conditions.

So far, more than 30 Atg proteins have been found to participate in the execution of autophagy. The formation of autophagosomes is considered to be regulated by two macromolecular complexes, the ULK1 complex (ULK1/Atg1-mTOR-Atg13-RB1CC1/FIP200), which is responsible for the initiation of autophagy, and the PtdIns3K complex (PIK3C3/VPS34- Beclin 1-Atg14), which is responsible for the nucleation of autophagy (Xie et al., 2015). ULK1 kinase can also recruit PtdIns3K complex by phosphorylating some of the components, which results in the production of phosphatidylinositol 3-phosphate [PI(3)P] and favors autophagosomal membrane nucleation (Russell et al., 2013). In addition, Atg5 and Atg12 have been showed to cooperate with Atg7, forming the Atg5-Atg12-Atg16-like 1 (Atg16L1) complex, which facilitates the elongation and expansion of autophagosome membranes to form a completely closed autophagosome (Nakatogawa, 2013), while the formation of microtubule-associated protein 1 light chain 3 (LC3)-phosphatidyl ethanolamine (PE) conjugate is also required in this process. Fujita et al. proposed that the Atg16L1 complex may function as a scaffold for LC3 lipidation and affect the sites of autophagosome synthesis (Fujita et al., 2008). Finally, the fusion of autophagosomes with lysosomes is also well-regulated, wherein hairpin-type tail-anchored SNARE syntaxin 17, pleckstrin homology domain containing protein family member 1 (PLEKHM1), and Atg14 have been identified as important regulators.

In addition, several complicated signaling pathways could also play important roles in autophagy regulation (Behrends et al., 2010; Mizushima and Komatsu, 2011). For example, the mTOR complex 1 (mTORC1) and AMP-activated protein kinase (AMPK), are well-known upstream regulators of autophagy. In nutrient-rich conditions, mTORC1 is overactivated, which then suppresses autophagy by directly binding and phosphorylating ULK1 (Hosokawa et al., 2009). While in nutrient-depleted conditions, AMPK is upregulated, which promotes the activation of ULK1 kinase complex by inactivating mTORC1 or dephosphorylating ULK1 (Egan et al., 2011), the activated ULK1 then induces Atg13 phosphorylation and autophagy.

BECN1 (beclin 1) is well-known as a key autophagy modulator; its effects depend on its binding proteins. For example, BECN1, which binds to core components of the Class III PI3K complex, promotes the formation of autophagosomes (Liang et al., 1999; Kihara et al., 2001). The BH3 structure of the BECN1, which binds to the antiapoptotic protein Bcl-2/Bcl-xl, inhibits the occurrence of autophagy, while downregulating Bcl-2 activates the autophagic pathway (Lian et al., 2011; Yang and Yao, 2015).

Phosphatidylinositol 3-kinase (PI3K) is another important regulator involved in phagosome maturation and autophagy (Thi and Reiner, 2012), which can be divided into three classes. Class I PI3K has been shown to inhibit autophagy through the PI3K-Akt-TSCl/TSC2-mTOR pathway (Hawkins et al., 2006; Pilli et al., 2012), and S14161, a Class I PI3K inhibitor, induces autophagy by regulating the Beclin-1/Vps34 complex (Wang et al., 2017). Besides, apelin-13, an adipokine, inhibit foam cell formation by activating autophagy via the Class III PI3K/Beclin-1 pathway (Yao et al., 2015).

The ubiquitin-binding protein, p62, also known as sequestosome1 (SQSTM1), is also involved in autophagy. Conventionally, p62 is considered as a cargo receptor recruiting/sequestering the ubiquitinated cargo to target autophagosomes and then degrade within lysosomes. This process can be suppressed by the Class III PI3K inhibitors, or consumption of the Atg12 protein homolog (Kim et al., 2008). Recently, it has also been found to play a more complicated role via regulating various signaling pathways including the mTORC1 pathway (Moscat et al., 2016); p62 deficiency impairs the translocation of mTORC1 to the lysosomes and its activation in response to amino acids and Tsc1 ablation (Duran et al., 2011; Umemura et al., 2016).

### The Dual Effects of Autophagy in Ischemic Stroke

The induction of autophagy has been identified in neurons, glia cells, brain microvascular cells, and other cell types after ischemic stroke (Wang P. et al., 2018). It is generally believed that autophagy can play a dual role in ischemic stroke (Table 2); moderate activation of autophagy could enable neuronal cell survival, while excessive autophagy triggers neuronal death. For example, mitophagy can induce mitochondrial clearance and the inhibition of apoptosis, which represents the neuroprotective effect of autophagy (Zhang et al., 2014; Shen et al., 2017). Sirtuin3 (Sirt3), upregulated by oxygen and glucose deprivation, increases autophagy through regulating the AMPK-mTOR pathway, and then plays a protective role in neuronal ischemia (Dai et al., 2017). Metastasis-associated lung adenocarcinoma transcript 1 (MALAT1) long non-coding RNA (lncRNA) also activates autophagy and protects against cerebral ischemia by binding to miR-200c-3p and upregulating Sirt1 expression (Wang et al., 2019). On the other hand, excess autophagy also contributes to endothelial damage and destruction of the blood–brain barrier (BBB) under ischemic conditions. Activation of the autophagy-lysosomal pathway after ischemia promotes degradation of the BBB component claudin-5, while the inhibition of autophagy prevents damage to brain microvascular endothelial cells during reperfusion (Yang G. et al., 2018). Similarly, the absence of the circadian clock protein period1 (PER1) suppresses hippocampal autophagy and leads to vulnerability during ischemic stroke (Rami et al., 2017). So far, modulations of autophagy intensity have been reported as feasible strategies in the treatment of ischemic stroke. Drugs such as dexmedetomidine have been found to protect neurons from ischemic damage by promoting autophagy (Luo et al., 2017), and some non-coding RNAs targeting autophagy have also been shown to play important roles in ischemic stroke (Wang N. et al., 2018; Yu et al., 2019).

**TABLE 2:** Autophagy and ferroptosis in ischemic stroke.

References	Interventions	Subjects	Targets	Effects
**Harmful autophagy**				
Feng et al. (2017)	Melatonin	MCAO mice	ER stress ↓	Melatonin protects against cerebral ischemia through inhibiting ER stress-dependent autophagy.
Bu et al. (2014)	W007B	MCAO rats	Beclin-1, LC3B-II ↓ p62 ↑	W007B protects against cerebral ischemia through inhibiting autophagy.
Baek et al. (2014)	Carnosine	MCAO rats	LC3-II formation ↓ P-p70S6K, p-mTOR ↑	Carnosine protects against cerebral ischemia at least partially by attenuating deleterious autophagy.
Jiang et al. (2017)	NaHS	MCAO rats	LC3 II/I ↓ P62 ↑ Autophagolysosomes ↓	NaHS protects against cerebral ischemia by inhibiting overactivated autophagy.
Li et al. (2012)	TMEM166 siRNA	MCAO rats	Beclin-1, LC3 ↓	TMEM166 siRNA protects against cerebral ischemia by inhibiting TMEM166-induced autophagy.
Luo et al. (2017)	DEX	MCAO mice, OGD primary cultured neurons	Bcl-1, p62, HIF-1α↑ LC3, Beclin-1 ↓	DEX protects against cerebral ischemia via inhibition of neuronal autophagy by upregulation of HIF-1α.
Yang G. et al. (2018)	HSYA	OGD primary BMECs	Autophagosomes ↓ LC3, Beclin-1 ↓ P-Akt, p-mTOR ↑	HSYA protects against OGD by inhibiting autophagy via the Class I PI3K/Akt/mTOR signaling pathway.
**Protective autophagy**				
Qi et al. (2015)	RIC	MCAO rats	P-Bcl-2 ↑ Bcl-2/Beclin1 complex ↓	RIC triggers autophagy and reduces mitochondrial damage after cerebral ischemia
Li et al. (2014)	Rapamycin	MCAO rats	LC3-II and Beclin-1 in the mitochondria ↑ P62 translocation to the mitochondria ↑	Rapamycin attenuates mitochondrial dysfunction following cerebral ischemia, which is linked to enhanced mitophagy.
Zhang et al. (2014)	TM and TG	MCAO mice	ER stress ↑	TM and TG protects against cerebral ischemia via inducing ER stress, which are based on the PARK2-mediated mitophagy.
Wang et al. (2014b)	ARRB1	OGD neurons	Autophagosome ↑	ARRB1 protects against OGD through coordination of BECN1-dependent autophagy.
Dai et al. (2017)	/	OGD neurons	Beclin-1, LC3-II ↑ P-AMPK ↑ P-mTOR ↓	Sirt3 protects against OGD by inducing autophagy through regulation of the AMPK-mTOR pathway.
Gao et al. (2015)	IPC	OGD neurons	P-Akt, LC3-II/LC3-I↑	IPC may attenuate ischemic injury in neurons through induction of Akt-independent autophagy.
Shen et al. (2017)	APC	MCAO mice OGD neurons	TOMM20, COX4I1 ↓ PARK2 translocation to the mitochondria ↑	PARK2-induced mitophagy is required for the APC-mediated neuroprotection to ischemic injury, which also extends the reperfusion window of cerebral ischemia.
Wang et al. (2019)	MALAT1 lncRNAs	OGD BMECs	MALAT1, Sirt1 ↑ MiR-200c-3p ↓	MALAT1 lncRNAs activates autophagy and protects against OGD by binding to miR-200c-3p and upregulating Sirt1 expression.
**Ferroptosis**				
Ahmad et al. (2014)	SES	MCAO mice	GSH ↑ MAPK/ERK, P38 ↓ Superoxide radical ↓ Lipid peroxidation ↓	SES induces neuroprotection by ameliorating lipid peroxidation and increased GSH activity following cerebral ischemia.
Guan et al. (2019)	Carvacrol	OGD neurons Gerbils with bilateral carotid artery ligation	Lipid peroxide ↓ Gpx4 ↑	Carvacrol protects against ischemic stroke by inhibiting ferroptosis through increasing the expression of Gpx4.
Alim et al. (2019)	Selenium	MCAO mice	TFAP2c, Sp1 ↑ Gpx4 ↑	Pharmacological Se supplementation protects cells from ferroptosis in ischemic stroke via increasing GPX4 expression.
Liu et al. (2017)	DMED	OGD PC12 and primary neuronal cells	SOD, GSH-Px ↓	DMED protects against OGD depending on its anti-oxidative activity.
García-Yébenes et al. (2012)	Iron-fed diet	MCAO mice	Iron accumulation ↑	Iron-fed diet increases ischemic damage and HT by increasing brain iron accumulation.
Hanson et al. (2009)	DFO	MCAO rats	Iron accumulation ↓	Intranasal DFO treatment decreases infarct volume by inhibiting iron overload.
Tuo et al. (2017)	/	Tau^–/–^ young and aged MCAO mice and rats	Intensity of iron accumulation	Tau suppression induced by cerebral ischemic prevent ferroptosis in young tau-/- mice, while the protective benefit of tau-/- was negated in older mice due to the accelerated age-dependent brain iron accumulation.
García-Yébenes et al. (2018)	Iron-fed diet	Mice subjected to thromboembolic stroke treated with tPA	Lipid peroxidation ↑ Iron accumulation ↑	Iron-fed mice show less neuroprotection after tPA administration. Iron overload also exacerbates the risk of HT after early tPA administration enhanced basal serum lipid peroxidation.
Lan et al. (2020)	NTE	MCAO rats	TFR1, DMT1 ↓ Iron accumulation ↓ SLC7A11, Gpx4, GSH ↑	NTE treats ischemic injury by inhibiting ferroptosis through the TFR1/DMT1 and SCL7A11/Gpx4 pathways.
DeGregorio-Rocasolano et al. (2018)	ATf	MCAO rats OGD primary neuronal cells	TSAT, HTf uptake ↓ 4-HNE ↓	ATf reduces neuronal damage by preventing NMDA-induced HTf uptake and ROS production.
Papazisis et al. (2008)	DFO	Neonatal rats with hypoxia-ischemia	Iron accumulation ↓ Glutamate, aspartate ↓	DFO decreases the excitatory amino acid levels and improves the histological outcome after hypoxia-ischemia.
Millan et al. (2007)	/	Patients with acute ischemic stroke treated with tPA	Intensity of iron accumulation	Increased body iron stores are associated with poor outcome and symptomatic HT. Iron overload may offset the beneficial effect of thrombolytic therapies.
Pekcec et al. (2013)	/	MCAO mice	Sema3A, 12/15-LOX ↑	Sema3A increases cortical damage, which is reversed by 12/15-LOX inhibition.
van Leyen et al. (2006)	Baicalein	MCAO mice	12/15-LOX ↓	Baicalein protects against cerebral ischemia by inhibiting the 12/15-LOX pathway.
Yigitkanli et al. (2013)	LOXBlock-1	MCAO mice	12/15-LOX ↓	LOXBlock-1 protects against ischemic stroke by inhibiting lipid peroxidation.

**MCAO, middle cerebral artery occlusion; ER, Endoplasmic reticulum stress; NaHS, sodium hydrosulfide; TMEM166, transmembrane protein 166; RIC, remote ischemic conditioning; TM, tunicamycin; TG, thapsigargin; ARRB1, arrestin, β1; OGD, oxygen-glucose deprivation; Sirt1, sirtuin1; IPC, ischemic preconditioning; LC3, light chain 3; APC, acidic post-conditioning; MALAT1, metastasis-associated lung adenocarcinoma transcript 1; LncRNAs, long non-coding RNAs; BMEC, brain microvascular endothelial cell; PER1, PERIOD1; DEX, dexmedetomidine; HIF-1α, hypoxia-inducible factor-1α; HSYA, hydroxysafflor yellow A; SES, sesamin; GSH, glutathione; GSH-Px, glutathione peroxidase; Gpx4, glutathione peroxidase 4; DFO, deferoxamine; DMED, dexmedetomidine; SOD, superoxide dismutase; ROS, reactive oxygen species; HT, haemorrhagic transformation; NTE, naotaifang extract; TFR1, transferrin receptor 1; DMT1, divalent metal transporter 1; SLC7A11, solute carrier family 7 member 11; ATf, apotransferrin; TSAT, transferrin saturation; HTf, holotransferrin; tPA, tissue plasminogen activator; 12/15-LOX, 12/15-lipoxygenase.**

## Ferroptosis and Ischemic Stroke

**FIGURE 1:**
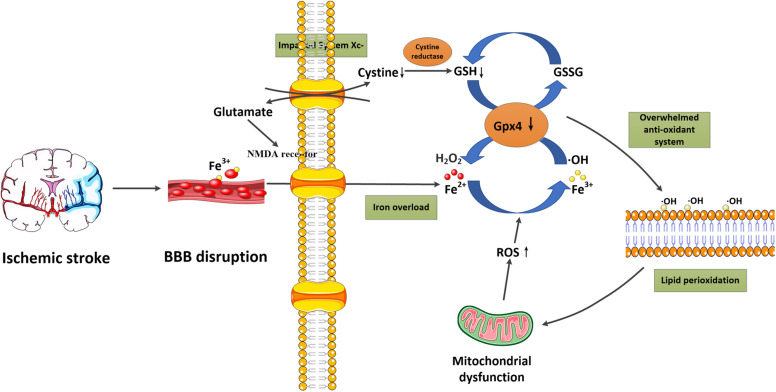
Schematic overview of ferroptosis in ischemic stroke. Following ischemic stroke, the BBB is disrupted, which allows a large amount of ferritin-containing Fe^3+^ in the blood to be released into the brain parenchyma. This converts hydrogen peroxide to hydroxyl radicals via the Fenton reaction. Meanwhile, system Xc- is simultaneously impaired, which inhibits cystine-glutamate exchange and decreases the generation of the antioxidant GSH, and reduces its oxidation to GSSG catalyzed by Gpx4. Furthermore, excessive glutamate accumulation within the cell can also activate glutamate-NMDA receptors, which in turn promote neuronal iron uptake. These two processes consume antioxidants and result in oxidative stress. The excessive free radicals then target sensitive fatty acids to promote lipid peroxidation, leading to the impaired integrity of lipid membranes and mitochondrial dysfunction, which can release ROS into the cytoplasm. BBB, blood-brain barrier; Gpx4, glutathione peroxidase 4; GSH, glutathione; GSSG, glutathione disulfide; NMDA, N-methyl-D-aspartate; ROS, reactive oxygen species.

Ferroptosis is a recently discovered regulated form of cell death based on iron-dependent lipid peroxidation. In general, the induction of ferroptosis can be divided into four critical events: (1) iron overload, (2) glutathione (GSH) depletion and glutathione peroxidase 4 (Gpx4) inactivation, (3) lipid peroxidation, and (4) impaired system Xc-. These events form positive feedback loops and generally push cells toward death. Stopping any critical events would stop the co-dependent events and then suppress the occurrence of ferroptosis. We now briefly introduce the relationship between these events and their potential roles in ischemic stroke (Figure 1 and Table 2).

### Iron Overload

It is well-known that iron metabolism plays an important role in the brain. In general, iron-loaded transferrin (holotransferrin) transports iron to the brain through the endothelial cells of the BBB and the choroid plexus epithelium. Physiologically, the brain can be sheltered from fluctuations in systemic iron due to the protection provided by the BBB. However, under acute ischemic conditions, the BBB is disrupted, which allows the entry of free iron and ferritin into the brain parenchyma, converting hydrogen peroxide to hydroxyl radicals via the Fenton reaction (Kell, 2009; Chen et al., 2011). This process significantly increases the generation of reactive oxygen species (ROS), which promotes nucleic, proteomic, and membrane damage, and finally mediates ferroptotic cell death (DeGregorio-Rocasolano et al., 2019). Nowadays, iron overload has been identified as a major source of oxidative stress in ischemic brains (Carbonell and Rama, 2007). Furthermore, in the early stage of reperfusion, it also increases the risk of hemorrhagic transformation, and thereby exaggerates the poor outcomes associated with cerebral ischemia (García-Yébenes et al., 2018). In clinical studies, high levels of serum ferritin also offset the beneficial effect of thrombolytic therapies in ischemic stroke patients (Millan et al., 2007). These studies confirm the important role of iron overload in ischemic stroke.

### Depletion of GSH and Gpx4 Inactivation

Glutathione is a tripeptide (Glu-Cyc-Gly) that can combine with free radicals to protect cells from oxidative damage (Aoyama and Nakaki, 2015). Gpx4 is an important antioxidant enzyme that converts GSH into oxidized glutathione (GSSG), and then transforms the cytotoxic lipid peroxides to the corresponding alcohols. During ferroptosis, accumulation of redox-active iron consumes GSH reserves through the Fenton reaction, which then suppresses the activity of Gpx4 and leads to an overwhelming antioxidant response (Nunez et al., 2012). The absence of antioxidant enzymes in turn result in the accumulation of iron. In a mouse model of ischemic stroke, decreased GSH and Gpx4 activity in neurons have been identified, accompanied with enhanced lipid peroxidation (Ahmad et al., 2014). A study using brain cells also indicated that a reduction of GSH can sensitize cells to oxidative stress and trigger lipid peroxidation (Andersen et al., 1996). In a gerbil cerebral ischemia model, carvacrol also successfully protects hippocampal neurons against ferroptotic cell death by increasing the expression of Gpx4, which provides a promising target for ischemic stroke therapies (Guan et al., 2019).

### Lipid Peroxidation

The depletion of GSH, as well as Gpx4 inactivation, has been confirmed as a requisite factor for the promotion of lipid peroxidation during ferroptosis. Specifically, when the antioxidant system is overwhelmed due to the iron overload, excessive free radicals target sensitive fatty acids and promote their peroxidation, which then impairs the integrity of lipid membranes and induces suicide signaling cascades (Reed, 2011; Yin et al., 2011). Besides, it also causes lysosomal membrane permeabilization and the release of redox-active iron into the cytoplasm, which in turn promotes the generation of Fenton radicals, cell membrane denaturation, and GSH depletion (Galluzzi et al., 2014). During this step, peroxidation of polyunsaturated fatty acids (PUFAs) has been considered as a key player (Kagan et al., 2017). Two enzymes, acyl-CoA synthetase long chain family member 4 (ACSL4) and lysophosphatidylcholine Acyltransferase 3 (LPCAT3), have been identified to be responsible for the biosynthesis and remodeling of PUFA-containing phospholipid, while inhibiting ACSL4 or LPCAT3 prevents ferroptotic cell death (Yuan et al., 2016; Li et al., 2019; Cheng et al., 2020). Then, oxidation of PUFA-phosphatidylethanolamines by cyclooxygenases, lipoxygenases (LOX) and cytochromes P450 lead to the accumulation of peroxides, finally contributing to the generation of lipid peroxides (Yang et al., 2016; Çolakoğlu et al., 2018; Tyurina et al., 2018). So far, inhibiting lipid peroxidation through lipoxygenase inhibitors or lipophilic antioxidants has been shown successfully reduce ferroptotic cell death (Matsushita et al., 2015). Since membrane phospholipids in the brain are highly enriched in PUFAs, they are easily disrupted by a high quantity of ROS, and thereby induce lipid peroxidation (Chen et al., 2008). In animal and human ischemic stroke models, it has been found that lipoxygenase inhibitors can play protective roles by eliminating the overexpression of lipoxygenases (Cui et al., 2010; Karatas et al., 2018). Besides, the increased 12/15-LOX was also observed following ischemic stroke, contributing to neurological damage, while 12/15-LOX inhibition reversed the detrimental effects (Pekcec et al., 2013; Yigitkanli et al., 2013).

### Impaired System Xc-

Glutamate-induced neurotoxicity is a well-known important mechanism underlying ischemic stroke. In recent years, it was also identified to participate in the process of ferroptosis (Dixon et al., 2012). Non-synaptic extracellular glutamate in the brain is mainly derived from the system Xc-, which is responsible for glutamate extracellular export and cystine import (Domercq et al., 2007). In physiological conditions, system Xc- could maintain a reducing extracellular environment, but in ischemic stroke, excessive glutamate inhibits cystine uptake via inhibiting system Xc- (Banjac et al., 2008), which impairs cystine absorption and decreases the generation of antioxidant GSH (Conrad and Sato, 2012). In addition, the accumulated glutamate within the cell also results in the activation of glutamate-NMDA receptors, which promote neuronal iron uptake and eventually cause ferroptotic damage (Cheah et al., 2006). In neuronal cell lines, it has been shown that both the 5 -LOX inhibitor zileuton and the iron chelator ferrostatin-1 can protect neurons from glutamate-induced oxidative stress, through the inhibition of ferroptosis (Liu et al., 2015).

## Crosstalk Between Autophagy and Ferroptosis

**FIGURE 2:**
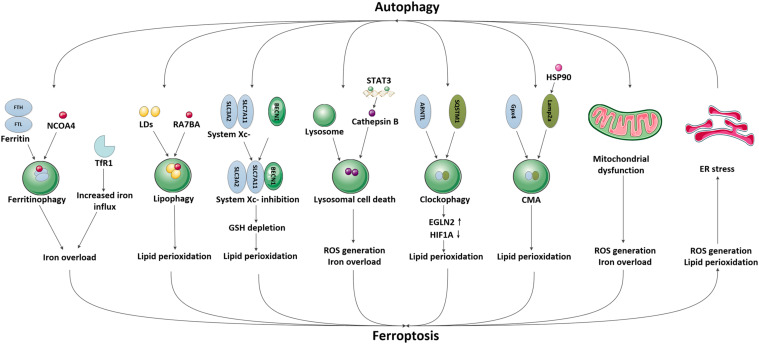
The possible pathways mediating the crosstalk between autophagy and ferroptosis in ischemic stroke. We have illustrated the possible pathways involved in the crosstalk between autophagy and ferroptosis and their downstream effects in ischemic stroke, including NCOA4-mediated ferritinophagy and upregulation of TfR1, RAB7A-mediated lipophagy, BECN1-mediated system Xc- inhibition, STAT3-mediated lysosomal cell death, SQSTM1-mediated clockophagy, HSP90-mediated CMA, mitochondrial dysfunction and ER stress.

In recent years, emerging studies have identified that some selective autophagy can also degrade damaged mitochondria, aggregated proteins, excess peroxisomes and invading pathogens through recognizing specific cargos, thus allowing the maintenance of intracellular homeostasis (Kraft et al., 2009; Rogov et al., 2014; Khaminets et al., 2016). These autophagic degradation may contribute to iron overload or lipid peroxidation, eventually causing ferroptosis (Kang and Tang, 2017; Zhou et al., 2019; Liu et al., 2020). Since the selective autophagy and ferroptosis have been found to play important roles in ischemic stroke, it is possible that autophagy may participate in the modulation of brain iron accumulation and lipid peroxidation following ischemic process, subsequently promoting ferroptotic cell death. A specific introduction to this hypothesis is identified in Figure 2.

### Nuclear Receptor Coactivator 4 (NCOA4)-Mediated Ferritinophagy and Regulation of TfR1

Iron is necessary for the high metabolic demands of brain cells, however, excessive free iron can promote oxidative stress and cause brain damage; thus, iron availability must be strictly controlled within brain cells. In general, cellular iron is stored in the non-toxic and bioavailable form of ferritin, which is composed of ferritin heavy chains (FTH) and ferritin light chains (FTL) (Arosio et al., 2009), which can prevent harmful oxidative stress when free iron is overloaded, and release iron when cells require it. The primary pathway to release iron from ferritin is via ferritinophagy, which is NCOA4-mediated ferritin degradation. Specifically, NCOA4 links ferritin to growing autophagosomal membranes, where ferritin is degraded and releases chelated iron, which is then used by the cell to induce oxidative stress (Mancias et al., 2014). Several studies have demonstrated that ferritin degradation and cellular free iron overload, induced by the overexpression of NCOA4, can promote ferroptosis, whereas the depletion or inhibition of NCOA4 increases serum ferritin levels and reduces free iron levels, which thereby inhibits oxidative stress during ferroptosis (Mancias et al., 2015; Gao et al., 2016; Hou et al., 2016). Moreover, the level of GSH is also increased with NCOA4 knockdown and is decreased with NCOA4 overactivation. These results strongly support the presence of a connection between NCOA4-induced ferritinophagy and ferroptosis through the regulation of iron homeostasis and GSH levels.

To date, little is known about the expression of NCOA4 in the human brain, however, it’s expression in murine and rat brains has been identified (Siriett et al., 2006; Kollara and Brown, 2010). Acute systemic consumption of NCOA4 shows accumulation of FTH1 within a week, which suggest a potential role of NCOA4 for FTH1 turnover in the brain (Santana-Codina et al., 2019). Interestingly, excessive iron measured as serum ferritin was also associated with poor prognosis following ischemic stroke (García-Yébenes et al., 2012), indicating the important role of ferritin as an iron carrier to mediate iron storage and release under ischemic conditions. Nowadays, a possible link between neurodegeneration and NCOA4-mediated ferritinophagy have been exhaustively reviewed (Quiles Del Rey and Mancias, 2019), however, their effects in ischemic stroke are still unclear. Since the disruption of iron homeostasis is concurrently observed with autophagy defects in ischemic stroke, and autophagy maintains cell homeostasis mainly by promoting the clearance of toxic proteins, we propose that it is highly possible for aberrant autophagy to mediate ferritin degradation, which then promote iron overload and ferroptosis in ischemic stroke.

Transferrin receptor 1 (TfR1) is a major receptor involved in iron transport to the brain, which plays an important role in maintaining the homeostasis of brain iron and regulating ferroptosis (Lo et al., 2007; Raje et al., 2007; Kawabata, 2019). The regulation of TfR1 expression as well as its related TfR/Tf endocytic pathway has been identified as a critical event that influences the outcome of ischemic stroke (Lo et al., 2007; Park et al., 2011; Lan et al., 2020). Interestingly, recent studies have shown that activation of autophagy increases the expression of TfR1 and subsequent intracellular iron (Qian et al., 2002). In wild-type cells, autophagy leads to ferroptosis via the degradation of ferritin and enhanced expression of TfR1, while in autophagy-deficient cells and autophagy inhibitor-treated wild-type cells, this effect is abolished (Park and Chung, 2019). These results raise the possibility that autophagy may regulate ischemic stroke by influencing the expression of TfR1 and iron-dependent ferroptosis.

### RAB7A-Mediated Lipophagy

Neutral lipids can deposit in the bilayer membranes of the endoplasmic reticulum (ER), which causes the outer layer to expand and form a unique dynamic organelle called lipid droplets (LDs) (Liu and Czaja, 2013). LDs have been found in most eukaryotic cells and in some prokaryotes, and can regulate cellular lipid storage and release in response to metabolic changes (Welte and Gould, 2017). Recently, the autophagic degradation of LDs, also known as lipophagy, has been identified to promote RSL3-induced lipid peroxidation and ferroptosis, while genetically increasing lipid storage by upregulating the level of tumor protein D52 (TPD52) suppressed lipid peroxidation and subsequent ferroptosis (Bai et al., 2019). In rat ischemic-reperfusion (I/R) models, studies have found a reshaping of neutral lipids and generation of LDs alongside the induction of lipophagy, which leads to lipid degradation (Lonati et al., 2019). Thus, it is possible that in ischemic stroke, the activated lipophagy might promote lipid peroxidation, which then contributes to ferroptosis.

### BECN1-Mediated System Xc- Inhibition

System Xc- is an amino acid anti-transporter composed of two core components: solute carrier family 7 member 11 (SLC7A11) and solute carrier family 3 member 2 (SLC3A2). It can exchange cystine and glutamate in and out of the cell; the imported cystine then reduces to cysteine and participates in the synthesis of the antioxidant GSH. So far, system Xc- has been shown to be involved in ischemic stroke through modulating glutamate transport and GSH synthesis (Krzyzanowska et al., 2016). In middle cerebral artery occlusion rat models, naotaifang extract treatment significantly inhibits ferroptosis by increasing the SLC7A11/GPX4 pathway (Lan et al., 2020). BECN1 is widely identified as an important autophagy modulator in ischemic stroke (Wang et al., 2014a,b; Liu et al., 2016). Recently, a study reported that BECN1 can also block the activity of system Xc- by directly binding to its core component, SLC7A11, and thereby promoting ferroptosis. In contrast, knockdown of BECN1, or inhibiting the phosphorylation of BECN1 limits the formation of a BECN1-system Xc- complex, which then suppresses ferroptosis induced by system Xc- inhibitors (Song et al., 2018). In primary oligodendrocytes, it has been shown that treatment with glutamate blocks system Xc- function, induces mitochondrial dysfunction, and promotes ferroptosis (Novgorodov et al., 2018). Besides, pretreatment with selenium in neurons attenuates glutamate toxicity, reduces ROS production, and preserves mitochondrial function after glutamate exposure and/or hypoxia, accompanied by reduced levels of BECN1 and LC3-II (Mehta et al., 2012). Since the dysfunction of system Xc- and BECN1 were concurrently observed with glutamate exposure, we propose whether BECN1 can interact with system Xc- to regulate ferroptosis in the brain. However, the upstream mechanisms to regulate BECN1 to determine its preferred interaction with system Xc- or Class III PI3K to mediate ferroptosis or autophagy remain undefined, which may be a key topic for future research.

### STAT3-Mediated Lysosomal Cell Death

Autophagy has been confirmed to result in the delivery of cytoplasmic contents and organelles to lysosomes for degradation. Recently, studies have found that lysosomal activity can also be impaired by ferroptosis, which provides a new hypothesis for the relationship between autophagy and ferroptosis. Since lysosomes are vulnerable to oxidative stress, they could be damaged by the intralysosomal Fenton reaction and the subsequent peroxidative instability of lysosomal membranes (Brunk et al., 1995; Ollinger and Brunk, 1995). In contrast, intralysosomal ferritin increases lysosomal stability via iron chelation, and then reduces oxidative stress (Garner et al., 1997, 1998). Besides, treatment with lysosome inhibitors has also shown to decrease erastin- and RSL3-induced ferroptotic cell death by inhibiting ROS production and intracellular iron overload (Torii et al., 2016). Signal transducer and activator of transcription 3 (STAT3) is a signaling molecule response to many cytokines and growth factors. It was recently identified that STAT3-mediated overexpression of cathepsin B significantly promotes ferroptosis via the activation of lysosomal cell death, whereas pharmacologically or genetically inhibiting STAT3 blocked ferroptotic cell death (Gao et al., 2018). These findings suggest a potential role of autophagy in ferroptosis via regulation of the lysosomal pathway. Nowadays, increased active cathepsin B levels and lysosomal membrane permeability have been proved to play important roles in ischemic stroke, while treatments reversing these impairments attenuated ischemic damage (Ni et al., 2018). However, the relationship between impaired lysosomal activity and ferroptotic damages in ischemic stroke requires further exploration.

### SQSTM1-Mediated Clockophagy

The circadian rhythm is an endogenous oscillation with a periodicity of about 24 h that is mainly regulated by circadian clock proteins, including aryl hydrocarbon receptor nuclear translocator-like protein 1/brain and muscle ARNT-like 1 (ARNTL/BMAL1) (Partch et al., 2014). It has been shown that the circadian rhythm plays an important role in maintaining normal internal cycles of behavior and brain physiology in human bodies, while its disruption can cause negative effects and even lead to vascular diseases, including ischemic stroke (Karatsoreos et al., 2011). In mouse I/R models, nighttime I/R injury has been found to cause less severe neuronal damage, which is related to the increased expression of circadian proteins such as BMAL1, PERI, and clock proteins (Beker et al., 2018). In human subjects, the outcome of ischemic stroke also shows a diurnal variation through the regulation of circadian clock proteins (Pardiwalla et al., 1993; Elliott, 1998). Besides, the autophagic machinery is inhibited in PER1^–/–^ hippocampal neurons, which may lead to vulnerability during cerebral ischemia, suggesting a functional relationship between autophagy and circadian rhythm (Rami et al., 2017).

In recent years, emerging studies have suggested that the circadian rhythm could control various cellular processes, including iron metabolism, oxidative stress, and cell death, which indicates its potential role in regulating ferroptosis (Magnone et al., 2014). Besides, the expression of circadian proteins also seems to be modulated by autophagy. A novel type of selective autophagy called clockophagy has been discovered, which is responsible for the degradation of the circadian clock protein ARNTL/BMAL1 via the cargo receptor SQSTM1/p62 (Yang et al., 2019). Early studies have reported that suppressing ARNTL expression by clockophagy effectively contributed to lipid peroxidation and ferroptotic cell death via upregulating the transcription of egl-9 family hypoxia-inducible factor 2 (EGLN2) and then decreasing hypoxia inducible factor 1 alpha (HIF1A)-dependent lipid storage. Genetically or chemically inhibiting ARNTL degradation or EGLN2 activation reduced ferroptosis, whereas destabilizing HIF1A promoted ferroptosis (Yang et al., 2019). These results provide a novel thought for the crosstalk between autophagy and ferroptosis through modulating circadian clock proteins, and prompts the possibility that the decreased expression of circadian clock proteins may lead to a poor prognosis in ischemic stroke due to clockophagy-induced ferroptosis.

### HSP90-Mediated CMA

Chaperone-mediated autophagy (CMA) is responsible for delivering certain cytosolic proteins with a pentapeptide CMA-targeting motif to lysosomes for degradation using molecular chaperones such as HSC70 (heat shock cognate protein 70) and LAMP2a (lysosome-associated membrane protein type 2a). In recent studies, scientists have found that CMA is highly activated during oxidative stress (Kiffin et al., 2004), which enhances the degradation of antioxidant proteins such as Gpx4 and then promote ferroptosis, while inhibition of CMA stabilized Gpx4 and protected against ferroptosis (Shimada et al., 2016; Muller et al., 2017; Zhu et al., 2017). Furthermore, a widely expressed heat shock protein, heat shock protein 90 (HSP90), which can be activated under oxidative stress (Carper et al., 1987; Sidera and Patsavoudi, 2014), has also been identified as an important molecular chaperone that can increase the levels of LAMP2a in the CMA pathway and mediate the degradation of Gpx4 during ferroptosis, while inhibition or knockdown of HSP90 blocks CMA and suppresses ferroptosis in HT-22 cells (a mouse neuronal cell line) (Wu et al., 2019). Since oxidative stress is a critical event during ischemic stroke, we assume that CMA might be activated under ischemic stroke, which then participates in ferroptosis by inducing Gpx4 degradation.

### Mitochondrial Dysfunction

Mitochondrial dysfunction is a major pathological process and also a critical therapeutic target in ischemic stroke. It is regulated by a complex machinery network, which forms a vicious cycle to disrupt mitochondrial homeostasis (Yang J. L. et al., 2018). Mitophagy is a special type of autophagy that can dictate mitochondrial turnover by degrading damaged mitochondria (Pickles et al., 2018). Recently, the activation of mitophagy has been shown to protect against mitochondrial damage in ischemic stroke, which indicates the important role of autophagy in regulating mitochondrial dysfunction (Shen et al., 2017; Yuan et al., 2017). In addition, ferroptosis has also been found to participate in the execution of mitochondrial dysfunction. Morphologically, ferroptosis is characterized by the atrophy of mitochondria with increased membrane densities, as well as reduced or even absent mitochondria crista and a ruptured outer membrane. Mechanically, as a core organelle to regulate iron metabolism, as well as substance and energy metabolism, the impairment of mitochondria can also affect cellular iron utilization and disrupt redox homeostasis, and then contribute to ferroptosis (Li et al., 2020; Wang et al., 2020). In neuronal cells, recent studies have reported that erastin- or RSL3-induced ferroptosis are associated with BID transactivation to mitochondria, increased mitochondrial fragmentation, and decreased ATP levels, while the inhibition of BID preserves integrity and function of the mitochondria and prevents ferroptosis (Neitemeier et al., 2017; Jelinek et al., 2018). Since the severity of ferroptosis is tightly associated with the disruption and recovery of mitochondria, while mitophagy is responsible for removing the damaged mitochondria and dictating mitochondrial turnover, we assume that the induction of mitophagy might be able to manipulate ferroptosis via regulating mitochondrial function. Notably, these findings also provide novel concepts regarding therapeutic interventions for ischemic stroke.

### ER Stress

The accumulation of misfolded proteins in ER, known as the unfolded protein response (UPR), disrupts ER homeostasis and leads to ER stress. Nowadays, ER stress has been confirmed as an essential factor mediating cell death in ischemic stroke (Nakka et al., 2010). Autophagy is a critical process activated by ER stress, which is responsible for removing misfolded proteins. In ischemic stroke, the induction of autophagy by ER stress can lead to two-sided effects. On the one hand, inhibition of ER stress-dependent autophagy could alleviate acute neuronal ischemic injury (Feng et al., 2017). On the other hand, salubrinal, an ER stress inhibitor, inhibits both the activation of autophagy and neuroprotection mediated by brain ischemic preconditioning (Gao et al., 2013). In contrast, activation of autophagy can also regulate ER stress. It has been shown that inhibition of autophagy by 3-Methyladenine (3-MA) significantly aggravates ER stress in ischemic stroke, while treatment with the autophagy inducer rapamycin reverses these effects (Sheng et al., 2012; Fan et al., 2016).

Recently, emerging studies have also identified the relationship between ER stress and ferroptosis (Dixon et al., 2014). For example, redox imbalance and lipid peroxidation can trigger ER stress (Vladykovskaya et al., 2012). RNA sequencing demonstrated that inhibition of system Xc- can lead to the activation of ER stress and upregulation of CHAC1 (ChaC, cation transport regulator homolog 1) (Dixon et al., 2014). Interestingly, ferroptosis could also share cell death pathways with autophagy via the ER stress response. Both ferroptosis inducers [artesunate (ART) and erastin (ERA)] and autophagy inducers [bortezomib (BOR) and XIE62-1004] promote the formation of autophagosome by regulating ER stress (Lee et al., 2020). These results not only provide a better understanding for the manipulation of ER stress, but also provide a new thought for the relationship between autophagy and ferroptosis in ischemic stroke.

### Other Potential Pathways Linking Autophagy and Ferroptosis

In addition to the above, there are some other signals that also indicate potential links between ferroptosis and autophagy. For example, autophagy can significantly decrease the levels of GSH (Mancilla et al., 2015; Stockwell et al., 2017), while both autophagy inhibitors and selective ferroptosis inhibitors improve GSH levels and suppress cell death, and vice versa (Desideri et al., 2012; Sun et al., 2018). These results suggest an important role of GSH in regulating both ferroptosis and autophagy, and that activation of one process might promote another through regulating GSH levels. ACSL4 is a critical enzyme involved in arachidonic acid (AA) metabolism and has been discovered to influence the sensitivity to ferroptosis (Yuan et al., 2016; Doll et al., 2017). Interestingly, it has also been identified as a novel activator of the mTOR pathway (Orlando et al., 2015). Since mTOR can protect cells from excess iron and ferroptosis (Baba et al., 2018), it might be a potential target for ACSL4 to modulate ferroptosis sensitivity. On the other hand, the induction of ferroptosis can also influence autophagy. For example, erastin-induced excessive ROS generation can activate autophagy, while overexpression of Gpx4 suppressed ROS-induced autophagy (Garg et al., 2013). Treatment with curcumin caused significant iron deprivation and then induced protective autophagy, while iron supplementation suppressed the occurrence of autophagy (Yang et al., 2017). Furthermore, the products of lipid peroxidation can also inhibit autophagy by activating mTORC1 signaling under ischemic conditions (Ma et al., 2011), or causing lysosomal dysfunction and lipofuscin generation to reduce autophagy activity (Krohne et al., 2010). Heat shock 70 kDa protein 5 (HSPA5) is an important molecular chaperone expressed primarily in the ER. It has been shown to effectively protect against cell death in response to ER stress-induced autophagy (Chang et al., 2019). Besides, a recent study also demonstrated that overexpression of HSPA5 can negatively regulate ferroptosis by limiting Gpx4 degradation and lipid peroxidation (Zhu et al., 2017). In the future, additional studies are needed to clarify whether the two processes mediated by HSPA5 function separately, or in cooperation with each other.

## Conclusion and Perspectives

Nowadays, a number of cell death pathways have been discovered (Galluzzi et al., 2014), which can cooperate with each other to help maintain organismal homeostasis. Clarification of their molecular mechanisms and crosstalk between each pathway would not only favor a comprehensive understanding of cell death pathways, but also open up new therapeutic approaches for related diseases. In recent years, the interrelationship between autophagy and ferroptosis has attracted more and more attention, which provides a novel concept regarding the regulation of cell death. However, their potential effects underlying ischemic stroke have not yet been discussed. In this review, we briefly summarize current knowledge on the mechanisms of autophagy and ferroptosis, while focusing on the possible pathways that mediate their crosstalk during ischemic stroke. Nevertheless, a lot of questions still existed before its clinical application. For example, the precise mechanisms underlying ferroptosis to govern iron and lipid metabolism in ischemic stroke remain to be explored, and the functional role of the different types of autophagy or the associated autophagy receptors in ferroptosis are still unclear. Besides, what are the effector molecules downstream of the two pathways to induce cell death? And can they influence each other and form a feedback loop in ischemic stroke? Moreover, since autophagy plays a dual role in ischemic stroke, it is critical for future interventions to manipulate the intensity of autophagy to find a balance between ferroptosis and autophagy and minimize neurological damages. Lastly, recent research has revealed that inhibition of two and more cell death pathways simultaneously can decrease ischemic stroke damage more significantly than inhibiting a single one (Tian et al., 2018). Therefore, interventions targeting both autophagy and ferroptosis at the same time could actually provide us with new ideas for the future treatment of ischemic stroke. All in all, there is still a long way to go before we fully understand the crosstalk between these two processes in ischemic stroke.

## Author Contributions

JL and Z-NG wrote the manuscript. X-LY, SH, YL, and J-XR prepared the figures. YY reviewed and edited the manuscript. All authors contributed to the article and approved the submitted version.

## Conflict of Interest

The authors declare that the research was conducted in the absence of any commercial or financial relationships that could be construed as a potential conflict of interest.
